# Primary pancreatic diffuse large B‐cell lymphoma, activated B‐cell subtype, diagnosed by endoscopic ultrasound‐guided fine needle aspiration—A case report and review of the literature

**DOI:** 10.1002/ccr3.3605

**Published:** 2020-12-04

**Authors:** Heather Jones, Jesse Qiao, Osvaldo Padilla, Attilio Orazi

**Affiliations:** ^1^ Paul L. Foster School of Medicine Texas Tech University Health Sciences Center El Paso El Paso TX USA; ^2^ Department of Pathology Paul L. Foster School of Medicine Texas Tech University Health Sciences Center El Paso El Paso TX USA; ^3^ The Hospitals of Providence, Transmountain Campus El Paso TX USA

**Keywords:** activated B‐cell, diffuse large B‐cell lymphoma, endoscopic ultrasound‐guided fine needle aspiration, pancreatic lymphoma, pancreatic mass

## Abstract

Although primary pancreatic lymphoma is a rare cause of pancreatic mass, this diagnosis should be considered during work‐up. Furthermore, when adequate diagnostic material is available from biopsy, complete workup of the lymphoma, including not only type but also subtype when applicable, should be performed.

## Graphical abstract

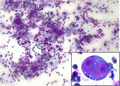

## 1 INTRODUCTION

Lymphomas rarely present as a primary pancreatic tumor and may pose diagnostic challenges due to limited material obtained by endoscopic ultrasound‐guided fine needle aspiration. We report a case of diffuse large B‐cell lymphoma of activated B‐cell subtype with associated clinical, radiological, and pathological findings and accompanying literature review.

The gastrointestinal tract is a common site for extranodal lymphomas, but primary pancreatic lymphoma (PPL) is rare and comprises only 1.3% to 1.5% of all pancreatic malignancies.^1^ Diffuse large B‐cell lymphoma (DLBCL) is the most common PPL, accounting for 77%‐80% of all cases.^2^, ^3^, ^4^ It may be difficult to clinically differentiate from other pancreatic neoplasms based solely on clinical presentation, laboratory results, and imaging tests.^1^ However, an accurate diagnosis of a pancreatic DLBCL is needed to determine appropriate treatment options and prognostic implications.^1^, ^5^ Furthermore, DLBCL can be subclassified by cell of origin, which includes germinal center B‐cell (GCB) and activated B‐cell (ABC) subtypes. The diagnosis of PPL is made by microscopy, as in other body sites through the similar use of morphology and immunohistochemistry. This diagnosis is helpful in understanding pathogenesis, prognosis, and adjuvant treatment options.^6^ We present a case of DLBCL that is made through fine needle aspiration of the pancreas to show how this diagnosis can be made, even though it is not commonly made by this type of sampling. We also utilized immunohistochemical stains to make a diagnosis of DLBCL of ABC subtype using the Hans algorithm,^7^ which is rarely reported in the literature for cytology specimens.

## 2 CASE HISTORY

**FIGURE 1:**
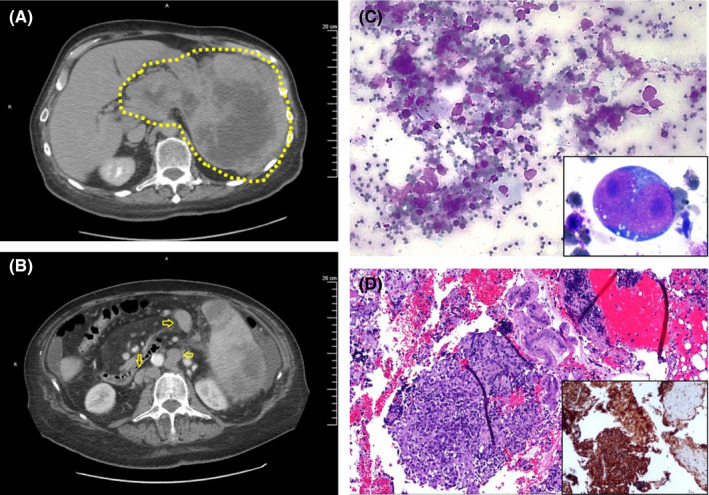
A, Abdominal CT scan with contrast shows a necrotic pancreatic mass extending into the spleen. It measures 18 cm in diameter and is encircled by the yellow dotted line. B, Abdominal CT scan also shows lymphadenopathy as noted by yellow arrow, which supports regional pancreatic lymph node involvement by the patient's DLBCL. C, Diff‐Quick‐stained FNA aspirate smear at 400× demonstrates discohesive and large atypical lymphoid cells on a background of necrotic debris and crush artifact. Some of the atypical lymphoid cells have nucleoli (overlaid image). D, H&E‐stained cell block at 100× shows abnormal proliferation of large atypical lymphoid cells. Contaminant normal gastric mucosa was also present. The majority of the abnormal lymphoid cells are positive for CD20, indicating B lymphoid lineage (overlaid image)

A 72‐year‐old man with no significant past medical history presented with epigastric pain and diarrhea for 2‐week duration. He also reported an unintentional weight loss over the previous 2 months. A CT scan of the abdomen demonstrated a large, primary necrotic mass in the tail of the pancreas with extension into the spleen (Figure 1A). There were also multiple enlarged lymph nodes present in the abdomen.

### 2.1 Differential diagnosis and investigations

Based on presentation and imaging, the differential diagnosis for this pancreatic mass included various pancreatic malignancies: carcinoma, lymphoma, and neuroendocrine tumor. The patient then underwent an endoscopic ultrasound‐guided fine needle aspiration (FNA) of the pancreatic mass. On‐site adequacy was performed by a cytotechnologist. The resulting cytological smears and cell block showed medium‐ to large‐sized, discohesive cells on a background of crush artifact and necrotic debris (Figure 1B and C). Initial impression of the mass was carcinoma, and therefore, no flow cytometry was performed. Immunohistochemical staining was performed using a cell block with the Benchmark XT instrument that included antigen retrieval, applying primary and secondary antibodies, and a horseradish peroxidase assay. These abnormal cells were found to be CD20(+) (Figure 1C), CD79a(+), and PAX‐5 (focal +). The Ki‐67 showed >75% nuclear positivity. Pan‐keratin, desmin, synaptophysin, CD30, Cyclin D1, CD10, c‐MYC, and EBV LMP were negative. BCL‐6, BCL‐2, and MUM‐1 were also positive, consistent with a DLBCL of ABC subtype.

B‐cell gene rearrangement studies by PCR for IgH/L chain gene rearrangement were also performed at a reference laboratory, which were positive for IgH/L chain gene rearrangement.

## 3 TREATMENT AND OUTCOME

After diagnosis, the patient received care at a tertiary care center and was lost to follow‐up at our facility. However, standard first‐line treatment for DLBCL of any subtype is rituximab, cyclophosphamide, doxorubicin, vincristine, and prednisone (R‐CHOP) or a similar chemotherapy regimen, as in other body sites.^5^, ^6^ However, determination of DLBCL subtype can be important for prognosis and adjuvant treatment options, with the ABC subtype carrying poorer prognosis than GCB.^5^, ^7^ Clinical follow‐up for progression or regression of disease is important, since patients with DLBCL, ABC subtype, as in this case, have a 5‐year progression‐free survival of 31% and overall survival of 45%.^8^

## 4 DISCUSSION

PPL is a rare neoplasm that typically is diagnosed by the sixth decade with a male predominance.^9^ It has been previously defined as a lymphoma that is localized to the pancreas with or without peripancreatic nodal involvement.^10^ However, the World Health Organization framework for primary extranodal lymphomas has refined this definition to include cases where the bulk of the tumor is localized to the pancreas.^11^ In our case, the tail of the pancreas was involved with extension to the spleen, which fulfills that criteria.

The most common histological subtype of PPL is DLBCL.^2^, ^3^, ^4^ Limited number of cases with or without further determination of subtype are reported in the literature, which were also made on pancreatic FNAs.^1^, ^2^, ^3^, ^4^ These tumors may be clinically and radiologically mistaken for carcinomas, neuroendocrine tumors, or other PPLs.^1^ PPL is an entity where surgical intervention is typically not necessary and specific chemotherapeutic protocols are utilized, similar to the treatment of DLBCL at other body sites. The pathological diagnostic criteria for DLBCL (as well as categorization by cell or origin) are similar in the pancreas, as in other sites. As a result, a correct diagnosis is paramount with adequate cell block material for additional studies (immunohistochemistry, flow cytometry, etc). This is enhanced by having an on‐site adequacy assessment by a cytotechnologist or pathologist at the time of the endoscopic ultrasound‐guided FNA procedure.

DLBCL can be further categorized by cell of origin (GCB vs ABC) on histologic sections by Hans algorithm, due to associated differences in gene expression, chromosomal aberrations, recurrent mutations, and survival differences.^7^ DLBCL of either cell of origin is typically involves similar treatment, but it has been shown that patients with ABC subtype have an inferior outcome following traditional chemoimmunotherapy, which is rituximab, cyclophosphamide, doxorubicin, vincristine, and prednisone (R‐CHOP).^5^ Clinical follow‐up is important, since treatment outcomes can be extremely variable, with approximately 60% of patients being cured with frontline chemotherapy, while the remaining patients may have refractory or relapsed disease with much poorer outcomes.^9^ However, in this particular case, the diagnosis and categorization by cell of origin was made by cytology, which is rarely reported in the literature. As a result, additional studies are needed to address the clinical utility of this categorization in cytological specimens.

In conclusion, pancreatic DLBCL with ABC subtype is a rare neoplasm, but a correct diagnosis is necessary for treatment options and prognosis. As shown by this case, an appropriate sample is needed in order to make a diagnosis of DLBCL of ABC cell type. In this case, it involved a pathologist being present at the time of FNA pancreatic procedure to evaluate the sample. As a result, the appropriate immunohistochemical stains and an adjunct molecular analysis were performed on an adequate cell block to help make a diagnosis.

## CONFLICT OF INTEREST

There are no conflicts of interest to disclose.

## AUTHOR CONTRIBUTIONS

HJ: performed data analysis, literature search, and manuscript preparation, editing, and review. JQ: performed concept, data acquisition and analysis, and manuscript editing and review. OP: performed concept, literature search, data analysis, and manuscript preparation, editing, and review. AO: performed concept, data analysis, and manuscript editing and review. The manuscript has been read and approved by all authors, and requirements for authorship have been met. Each author believes that the manuscript represents honest work.

## ETHICAL APPROVAL

The authors consciously assure for this manuscript that the material is the authors’ own work, has not been previously published or is currently being considered for publication elsewhere, reflects the authors’ own research and analysis, properly credits the meaningful contribution of authors, properly places results in context of prior and existing research, and properly discloses sources. All authors have been actively involved in substantial work leading to the paper and take responsibility for its content.

## Data Availability

Data sharing is not applicable to this case report type article as no new data were created or analyzed in this study.
